# Expression of HIF-2*α* and VEGF in Cervical Squamous Cell Carcinoma and Its Clinical Significance

**DOI:** 10.1155/2016/5631935

**Published:** 2016-06-20

**Authors:** Lixia Zhang, Qiang Chen, Jing Hu, Yue Chen, Chenglong Liu, Changshui Xu

**Affiliations:** ^1^Jiaxing Maternity and Child Health Care Hospital, Jianxin 314050, China; ^2^Department of Physiology, Basic Medical College, Nanchang University, Nanchang 330006, China; ^3^The First Clinical Medical College, Nanchang University, Nanchang 330006, China; ^4^The Second Clinical Medical College, Nanchang University, Nanchang 330006, China

## Abstract

CSCC is a systemic disease involving polygenic alteration and multiple steps, and HIF and VEGF are closely associated with tumorigenesis. Specimens surgically resected from 64 cases of CSCC and 22 cases of normal cervical tissue were selected randomly to detect the expression of HIF-2*α* and VEGF in CSCC for exploring their clinical significance; information regarding the age, lymph node metastasis, and FIGO staging were collected as well; expression of HIF-2*α* and VEGF was detected by qPCR and immunohistochemistry. We found that the expression of HIF-2*α* and VEGF mRNA in CSCC was significantly higher than that of normal cervical tissues and showed a positive correlation between them. The positive rates of HIF-2*α* and VEGF protein expression in CSCC and normal cervical tissues were 93.8% and 18.2%, respectively, with correlation between them. The expression of both HIF-2*α* and VEGF mRNA did not relate closely to age but the FIGO staging and lymph node metastasis. Compared with the counterpart control group, CSCC tissues with high FIGO staging and lymph node metastasis had a higher level of HIF-2*α* and VEGF mRNA expression. So, HIF-2*α* and VEGF were overexpressed in CSCC, which has a great clinical significance for its diagnosis.

## 1. Introduction

Cervical squamous cell carcinoma (CSCC) is a systemic disease involving polygenic alteration and multiple steps; expression of hypoxia-inducible factor (HIF) was crucial for the tumor to accomplish microangiogenesis successfully, which enabled rapid tumor proliferation with enough nutritional needs [1]. HIF-2*α* plays an important role in blood vessel growth, medullary hematopoiesis, energy metabolism, tumor genesis, and progression [2]. Vascular endothelial growth factor (VEGF) is one of the main cytokines by which the tumor stimulates angiogenesis [3] and which is capable of promoting angiogenesis in cervical squamous cell carcinoma [4]. In the present study, the expression of HIF-2*α*, VEGF genes, and proteins in CSCC was investigated, which explores their clinical significance.

## 2. Materials and Methods

### 2.1. Collection of Experimental Materials and Clinical Data

The study group comprised cancer tissues from 64 patients with proven cervical squamous cell carcinoma. All patients had undergone a resection at the Jiaxing Maternity and Child Health Care Hospital during January 2014 to August 2015. The median age at diagnosis was 51.3 ± 9.2 years (range: 38–70 years). All tumors were staged based on the new International Federation of Gynecology and Obstetrics (FIGO) classification from 2009. The number of patients in stage I, stage II, and stages III-IV is 30, 24, and 10, respectively. Twenty-two normal cervical tissues from cervical biopsy patients, age ranging from 32 to 36 (average age: 48.2 ± 8.7 years), were selected randomly as the controls. All patients did not undergo preoperative chemotherapy or radiotherapy and had no history of other tumors.

### 2.2. Real-Time Quantitative PCR (qPCR)

Firstly, 50–100 mg of tissue sample was taken in laminar flow cabinet, and the weighed tissue was placed on ice immediately and ground thoroughly with a mortar and pestle containing 1 mL of Trizol until the lysate was transparent. Then the lysate was decanted into a 1.5 mL EP tube and set at room temperature for 5 min. Next, 0.2 mL of chloroform was added to the tube which was then capped securely, shaken by vortex mixer for 15 seconds, and stood for 3 min in succession. Then, the tube was centrifuged for 15 min at 12000 rpm, 4°C, and the aqueous phase was transferred to a clean EP tube. Afterwards, 0.5 mL of isopropyl alcohol was added to the cleared lysate which was then mixed immediately and set at room temperature for 10 min. Following this, the tube was centrifuged for 10 min at 12000 rpm, 4°C, and then supernatant was discarded and 1 mL of 75% ethanol was decanted to wash the RNA pellet. Afterwards, the sample was mixed by vortexing and centrifuging for seconds. After a complete dissolution, the tube was centrifuged again for 5 min at 12000 rpm, 4°C, then the supernatant was discarded and the tube was let air-dry at room temperature for 5 min. Subsequently, 35 uL of diethyl pyrocarbonate (DEPC) was added to dissolve the RNA precipitation sufficiently. Lastly, the tube was stored at −80°C refrigerator. Meanwhile, a little sample was taken to detect purity and concentration of RNA by ultraviolet spectrophotometer.

After the isolation of fresh cervical specimens, tissue was cut out into pieces and put into a sterilized and enzyme-free Eppendorf and stored at −80°C refrigerator. Primers were designed using Primer Express software and were as follows: forward primer sequence genes and reverse primer sequence genes of HIF-2*α* were 5′-CATGCGCTAGACTCCGAGAACA-3′ and 5′-GCTTTGCGAGC-ATCCGGTA-3′, respectively, and the amplified fragment length was 94 bp; the forward primer sequence genes and reverse primer sequence genes of VEGF were 5′-GAGCCTTGCCTTGCTGCTCTA-3′ and 5′-CACCAGGGTCTCGATTGGATG-3′, respectively, and the amplified fragment length was 148 bp; forward primer sequence genes and reverse primer sequence genes of *β*-actin were 5′-TGGCACCCAGCACAATGAA-3′ and 5′-CTAAGTCATAGTCCGCCTAGAAGCA-3′, respectively, and the amplified fragment length was 186 bp. The total RNA was used as the template to synthesize cDNA at 37°C water bath for 1 h by employing the PrimeScript*™* reagent kit (Takara, Japan). Then PCR amplification and real-time quantitative analysis were performed. Specimens were subjected to 40 PCR cycles each consisting of 30 seconds of predenaturation at 95°C, 5 seconds of denaturation at 95°C, and 34 seconds of extension at 60°C. All the specific operations were done according to the instructions. Amplification sets reference gene group, target gene group, and control group. The relative expression level of HIF-2*α* and VEGF mRNA in the tissue was calculated by the cycles each reaction tube took to reach a given threshold according to the exponential amplification rule.

### 2.3. Immunohistochemistry

Fresh specimens were fixed with 4% Paraformaldehyde (PA) and embedded in Paraffin. Each case of Paraffin block was cut into 3 pieces of 4 um thick slices and stained with HE and immunohistochemistry, respectively. All of the specimens series included positive controls known to be positive for VEGF, and as for the negative control the primary antibody was replaced with PBS. Specific operations are summarized as follows: slices were deparaffinized and hydrated through a graded ethanol series and antigen retrieval was achieved by microwave and then the slides had undergone permeabilizing in 0.3% TritonX-100 for 15 min and were washed in PBS three times for 5 min. Sections were incubated for 10 min in 3% hydrogen peroxide and 60 min in working fluid of normal goat serum at room temperature successively and washed in PBS three times for 5 min, respectively. After that the sealing liquid was sucked out but wash was not permitted here. The sections were then incubated overnight at 4°C with primary antibodies (Abcam, Cambridge, UK; dilution, 1 : 100), washed with PBS three times for 5 min, and then incubated with secondary antibodies for 60 min at room temperature and washed in PBS three times for 5 min. Afterwards sections were incubated for 15 min in streptavidin-peroxidase (SP) in distilled water and washed in PBS three times at room temperature for 5 min. Diaminobenzidine (DAB) was added to the slides and then the sections were observed under microscope as they were staining for 3-4 min. The sections were then washed and humidified with ultrapure water to terminate staining and prevent dryness. After counterstaining with hematoxylin, differentiation with acidic alcohol, rehydration through a graded ethanol series, and transparentizing with dimethylbenzene, the specimens were finally mounted by Permount*™* mounting medium and observed under microscope.

*Result Estimation*. The positive materials were buffy particles mainly localized in the cytoplasm and partly in the nucleus or cell membrane. Classification standard: expression of HIF-2*α* and VEGF proteins was categorized quantitatively on the basis of the percentage of positive cells ≤5% (−), 5%~25% (+), 26%~50% (++), and >50% (+++). Examination of immunostaining was performed in a double-blinded fashion and the image under the microscope was observed and photographed by experienced pathologists in Jiaxing Maternity and Child Health Care Hospital.

### 2.4. Statistical Analysis

The relative mRNA expression of HIF-2*α* and VEGF gene was represented by $\overset{-}{x}\pm s$ and compared by independent-samples *t*-test using SPSS 17.0. Pearson correlation test was used for correlation analysis. As for the categorical data of HIF-2*α* and VEGF protein expression in the tissue, chi-square test was used for correlation analysis. The test standard of *α* is 0.05.

## 3. Results

### 3.1. Expression of HIF-2*α* and VEGF mRNA in CSCC and Correlation Analysis

**Figure 1:**
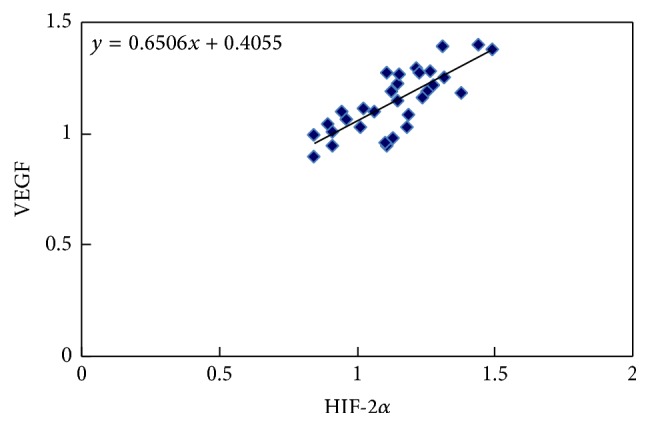
Pearson correlation test of the HIF-2*α* and VEGF mRNA expression in CSCC group. There was a significant correlation between the expression of HIF-2*α* and VEGF mRNA (Figure 1, *r* = 0.778, *p* < 0.001) at the 0.01 level (2-tailed).

According to real-time quantitative analysis, the expression levels of HIF-2*α* and VEGF mRNA in CSCC were 1.3418 ± 0.812 and 4.4543 ± 2.585, respectively, which were significantly higher than those of normal cervical tissue, 0.1375 ± 0.087 and 0.9127 ± 0.382, respectively (Table 1, *p* < 0.01). In our previous study, we also detected HIF-1*α* mRNA expression using qPCR method and found that there was no statistical significance between CSCC and normal cervical tissues (Table 1, *p* > 0.05). According to the result of Pearson correlation test, there was a significant correlation between the expression of HIF-2*α* and VEGF mRNA (Figure 1, *r* = 0.778, *p* < 0.001).

### 3.2. The Relationship between the HIF-2*α* and VEGF mRNA Expression in CSCC and Clinicopathological Parameters

After qPCR analysis, the expression of HIF-2*α* and VEGF mRNA was grouped according to age, FIGO stage, and lymphatic metastasis in CSCC group. According to the statistical analysis, there was no significant correlation between the expression of HIF-2*α* and VEGF mRNA and age (*p* > 0.05), but the expression of HIF-2*α* and VEGF mRNA was positively correlated with FIGO stage and lymph node metastasis (*p* < 0.05, Table 2).

### 3.3. The Protein Levels of HIF-2*α* and VEGF in CSCC and Normal Cervical Tissues and Their Correlation

**Figure 2:**
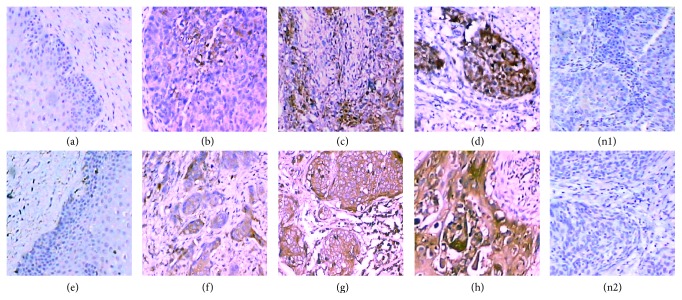
Protein levels of HIF-2*α* and VEGF in CSCC and normal cervical tissues (×200). HIF-2*α* expression in normal cervical tissues (−, (a)), CSCC (+, (b)), CSCC (++, (c)), CSCC (+++, (d)), and negative control (n1); VEGF expression in normal cervical tissues (−, (e)), CSCC (+, (f)), CSCC (++, (g)), CSCC (+++, (h)), and negative control (n2).

According to immunohistochemistry, the positive materials were buffy particles mainly localized in the cytoplasm and partly in the nucleus or cell membrane. The positive rates of HIF-2*α* and VEGF proteins in CSCC specimens were 93.8% (60/64) and 90.6% (58/64), respectively, which are significantly higher than that of normal cervical tissues, with a statistically significant difference (*p* < 0.01; see Table 3 and Figure 2).

**Figure 3:**
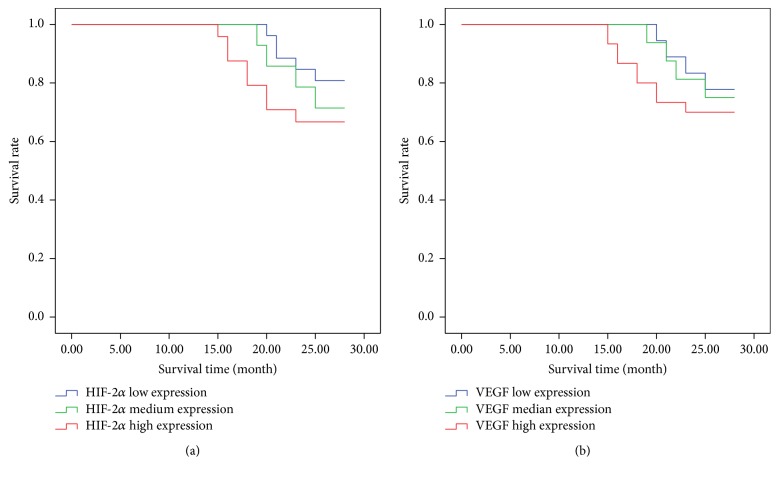
Effects of HIF-2*α* and VEGF expression on the survival rate of patients. (a) Survival rate of patients with high versus low HIF-2*α* and (b) survival rate of patients with high versus low VEGF.

At present, all patients had been followed up for 15~28 months. According to the results of immunohistochemistry, HIF-2*α* low expression group (−, +) was 26 cases (5 cases died and 21 cases survived), HIF-2*α* medium expression group (++) was 14 cases (4 cases died and 10 cases survived), and HIF-2*α* high expression group (+++) was 24 cases (8 cases died and 16 cases survived). Furthermore, we also found that VEGF low expression group (−, +) was 18 cases (4 cases died and 14 cases survived), VEGF medium expression group (++) was 16 cases (4 cases died and 12 cases survived), and VEGF high expression group (+++) was 30 cases (9 cases died and 21 cases survived). Based on the above results, we have made Kaplan Meier curves showing PFS/OS of patients with high versus low HIF-2*α* and VEGF, indicating that patients with high expression of HIF-2*α* and VEGF do worse in survival rates (Figure 3).

The rank correlation analysis of the HIF-2*α* and VEGF protein expression in CSCC showed that the positive levels of HIF-2*α* and VEGF protein expression had a positive correlation (Table 4, *r* = 0.514, *p* < 0.01).

## 4. Discussion

In the present study, we provided novel evidence regarding the expression of HIF-2*α* and VEGF which play a fundamental role in mediating hypoxia-induced tumor angiogenesis. We investigated HIF-2*α* and VEGF expression in CSCC tissue specimens using immunohistochemistry. Compared to the negative control, we found that the staining seen is specific to HIF-2*α* and VEGF, respectively. Staining was observed in the cytoplasm and nucleus of tumor cells. The results demonstrated that the positive rates of HIF-2*α* and VEGF proteins expression were significantly higher in CSCC tissue than in normal cervical tissue. We also investigated the expression of HIF-2*α* and VEGF at the mRNA levels which was analyzed by qPCR. The experiment results showed that there was a statistically significant correlation between FIGO stage and HIF-2*α*, VEGF mRNA expression levels. We also found that high mRNA expression of HIF-1*α* and VEGF was associated with increased metastatic potential in CSCC. We also made Kaplan Meier curves showing PFS/OS of patients with high versus low HIF-2*α* and VEGF and found that patients with high expression of HIF-2*α* and VEGF do worse in survival rates, deducing that there may be a similar association with grade and with progression-free survival/overall survival.

HIF, the most crucial heterodimeric transcription factor for hypoxia adaptation, is composed of *α* and *β* subunit. And *α* subunit is the functional and active one, which is highly regulated by oxygen [5]. In contrast to it, *β* subunit is the structural one, which is not regulated by oxygen. At present, *α* subunits which have been isolated and identified include 1*α*, 2*α*, and 3*α*. They can all combine with *β* subunits to form dimers and then assembled into HIF-1 protein, HIF-2 protein, and HIF-3 protein, respectively. The HIF-2*α* protein expression can be regulated by oxygen dependent degradation domain (ODD) under anoxic condition [6]. However, under normal oxygen concentration, HIF-2*α* is easy to be degraded by the ubiquitin-proteasome; thus its expression in the tissue or the cytoplasma remains at a low level. When the oxygen concentration in the cells is decreased, HIF-2*α* accumulates in the cytoplasma and then translocates to the nuclear due to the inhibition of the HIF-2*α* degradation process [7]. Afterwards, the HIF-2*α* combines with the *β* subunit in the nuclear and forms a dimer which can combine with the hypoxia-response element (HER) of the target gene specific series, and then it exerts its biological functions by activating the transcription of target gene [8]. The transcription factor family of HIF can modulate many genes with the decrease of oxygen concentration [9] and participates in the process of proliferation, metastasis, differentiation, and metabolism of tumor cell and angiogenesis [10, 11]. Therefore, HIF-2*α* may play a promoting role in the processes of occurrence, development, invasion, and metastasis of tumor. It has been found that there was less expression of HIF-2*α* protein in normal tissues and organs but it was expressed in many tumor cells (such as endometrial cancer, bladder cancer, renal cell carcinoma, breast cancer, liver cancer, and prostate cancer cells) [12]. In the present study, we detected both HIF-1*α* and HIF-2*α* mRNA expression using qPCR method and found that there was no statistical significance between CSCC and normal cervical tissues. On the contrary, the expression of HIF-2*α* mRNA in CSCC was statistically higher than that of normal cervical (*p* < 0.01), and VEGF was similar to it. Based on these results, we explored the relationship between HIF-2*α* and VEGF in CSCC. The positive rate of HIF-2*α* proteins in CSCC specimens detected by immunohistochemistry was also significantly higher than that of normal cervical tissues (*p* < 0.01). All the above results may be related to the hypoxia mechanism involved in the pathophysiologic process, for example, the self-renewal, malignant proliferation, and metastasis of tumor cells in CSCC [13, 14]. Some researches indicated that the family of HIF transcription factor maintained the growth and hypoxia adaptation of the tumor cells by modulating relevant target genes and participating in the energy metabolism procedure, and it was able to promote the expression of target genes like survivin to enhance the invasion ability of tumor cells [15]. From the clinical data, what further illustrated this point was that, in the present study, the expression level of HIF-2*α* mRNA in CSCC tissue of the patients with lymph node metastasis was found to be higher than that of counterpart control group.

VEGF is one of the major cytokines of tumor that can stimulate angiogenesis and the expression level of it increases with the decline of oxygen concentration in various types of cell. Thus, VEGF dependent and oxygen induced pathway of angiogenesis in the tissue is one of the crucial mechanisms of angiogenesis stimulated by tumor [3]. Plenty of malignant cells all have the ability of conducting VEGF autocrine, and the produced VEGF is able to induce the angiogenesis and cause mass production of new vessels that are aberrant in both structure and function, which accelerates the occurrence and development of tumor. Meanwhile, VEGF can enhance the viability of vascular endothelial cells, promote the mitosis, induce the renascence of capillary sprout, increase microvascular permeability, promote cell migration, and inhibit apoptosis [16, 17]. Lebrecht detected that there was a significant increase in the level of VEGF in the blood of invasive CSCC patients and the studies conducted by LEE showed that VEGF had an important role in enhancing the angiogenesis of CSCC as well [18]. In the present study, according to the qPCR, the expression level of VEGF mRNA in CSCC was found to be significantly higher than that of normal cervical tissues (*p* < 0.01), and the positive rate of VEGF proteins in CSCC specimens detected by immunohistochemistry was also significantly higher than that of normal cervical tissues (*p* < 0.01). All the results indicated that the expression level of VEGF was increased significantly in CSCC and they were consistent with the above studies.

In the previous studies, researchers found that the increased expression of HIF-1*α* contributed to the production of VEGF in tumor cells. Meanwhile, due to the structural similarity between HIF-1*α* and HIF-2*α*, HIF-2*α* may contribute to the production of VEGF in tumor cells as well [19]. In the present study, the expression of HIF-2*α* and VEGF mRNA in CSCC was found to be significantly higher than that of normal cervical tissues, with statistical significance (*p* < 0.05). From the results of mRNA and protein detection, there was a certain positive correlation between the expression of HIF-2*α* and VEGF (*r* = 0.778, *p* < 0.05; *r* = 0.514, *p* < 0.01). Kim and Kawanaka research groups successively found that HIF-2*α* was highly expressed in the tissues of CSCC patients and had something to do with radiotherapy sensitivity [20, 21]. Nevertheless, according to the present study, the expression of HIF-2*α*, VEGF mRNA did not relate closely to age (*p* > 0.05) but the FIGO staging and lymph node metastasis (*p* < 0.05). Some studies found that HIF-2*α* had more tumor specificities; for example, there was a low expression level of HIF-2*α* in small cell lung cancer (SCLC) patients which did not affect the survival of tumor cells [22], whereas the patients of nonsmall cell lung cancer (NSCLC) with a high expression of HIF-2*α* had poor prognosis and significantly shortened survival time [23], suggesting that HIF-2*α* may be an independent prognostic indicator. Other studies found that HIF-2*α* can combine with enhancers of VEGF gene more easily which is highly relevant to the expression of VEGF mRNA and that HIF-1*α* regulated tumor growth mainly by energy protection mechanisms under the hypoxia environment [24]. However, the growth promotion effects of HIF-2*α* were more helpful to vascular endothelial cell proliferation and abnormal hyperplasia of vessels in ischemic tissue [25, 26]. VEGF promoted division, proliferation, and migration of vascular endothelial cells specifically, which created new vessels to improve hypoxia of tumor to a certain extent. When the tumor encounter hypoxia, the high expression of HIF-2*α* accelerated the transcription of VEGF target genes and enhanced the stability of VEGF mRNA to accomplish the regulation of tumor angiogenesis. Besides, from some cell lines studies, HIF-1*α* and HIF-2*α* were reported to have specific effects in time and function, which meant that HIF-1*α* mainly mediated acute hypoxia adaptation, whereas HIF-2*α* mediated chronic hypoxia adaptation chiefly [27, 28].

## 5. Conclusions

In summary, the comprehensive evaluation results of present study showed that HIF-2*α* and VEGF are both involved in the process of CSCC. Moreover, HIF-2*α* probably participated in the processes of occurrence, development, invasion, and metastasis of CSCC by modulating the expression level of VEGF, while little HIF-2*α* and VEGF were expressed in the normal cervical tissues. In the present study, we investigated the change of the expression levels of HIF-2*α* and VEGF in CSCC tissues and discussed its probable pathogenesis and clinical significance according to clinical cases, which offered new ideas and theory to the prevention and treatment of cervical squamous cell carcinomas.

## Figures and Tables

**Table 1:** The mRNA levels of HIF-1*α*, HIF-2*α*, and VEGF in CSCC group and normal cervical tissues ($\overset{-}{x}\pm s$).

Group	Case number	HIF-1*α*	HIF-2*α*	VEGF
CSCC	64	1.1983 ± 0.302	1.3418 ± 0.812^a^	4.4543 ± 2.585^b^
Normal cervical tissue	22	1.1224 ± 0.522	0.1375 ± 0.087	0.9127 ± 0.382

^a^Compared with normal cervical tissues group, *t* = 11.672, *p* < 0.01. ^b^Compared with normal cervical tissues group, *t* = 10.628, *p* = 0.006.

**Table 2:** Expression of HIF-2*α* and VEGF mRNA in CSCC and clinicopathological parameters.

	Case number	HIF-2*α* mRNA level	VEGF mRNA level
Age			
≤40	10	1.337 ± 0.88^a^	4.276 ± 2.53^d^
>40	54	1.348 ± 0.85	4.532 ± 2.84
FIGO stage			
Stage I	30	1.242 ± 0.78^b^	3.284 ± 1.84^e^
Stage II	24	1.847 ± 0.93	5.352 ± 2.37
Stages III-IV	10	3.023 ± 1.13	7.125 ± 2.98
Lymphatic metastasis			
No	50	1.215 ± 0.84^c^	3.638 ± 1.76^f^
Yes	14	2.803 ± 1.12	6.421 ± 2.73

^a^Compared with >40 group, *t* = 0.05, *p* = 0.960.  ^b^Compared with other FIGO stages, *F* = 15.04, *p* < 0.01. ^c^Compared with yes group, *t* = 3.169, *p* < 0.01. ^d^Compared with >40 group, *t* = 0.367, *p* = 0.715. ^e^Compared with other FIGO stages, *F* = 12.81, *p* < 0.01. ^f^Compared with yes group, *t* = 3.615, *p* < 0.01.

**Table 3:** The protein levels of HIF-2*α* and VEGF in CSCC and normal cervical tissues.

Group	Case number	Immunohistochemical results				Negative (−)	Positive (+)	Positive rate (%)
		−	+	++	+++			
HIF-2*α*								
CSCC	64	4	22	14	24	4	60	93.8^a^
Normal cervical	22	18	4	0	0	18	4	18.2
VEGF								
CSCC	64	6	12	16	30	6	58	90.6^b^
Normal cervical	22	18	4	0	0	18	4	18.2

^a^Compared with normal cervical tissues group, *χ* ^2^ = 49.111, *p* < 0.01. ^b^Compared with normal cervical tissues group, *χ* ^2^ = 42.706, *p* < 0.01.

**Table 4:** Correlation analysis of HIF-2*α* and VEGF protein expression.

VEGF expression	HIF-2*α* expression		*χ* ^2^	*p*
	Negative (−)	Positive (+)		
Negative (−)	4	0	23.040	0.000
Positive (+)	6	54		

Chi-square test for correlation analysis, *χ* ^2^ = 23.040,  *r* = 0.514, *p* < 0.01.
