# Corrigendum to “Expression of HIF-2*α* and VEGF in Cervical Squamous Cell Carcinoma and Its Clinical Significance”

**DOI:** 10.1155/2017/5964107

**Published:** 2017-10-16

**Authors:** Lixia Zhang, Qiang Chen, Jing Hu, Yue Chen, Chenglong Liu, Changshui Xu

**Affiliations:** ^1^Jiaxing Maternity and Child Health Care Hospital, Jiaxing 314050, China; ^2^Department of Physiology, Basic Medical College, Nanchang University, Nanchang 330006, China; ^3^The First Clinical Medical College, Nanchang University, Nanchang 330006, China; ^4^The Second Clinical Medical College, Nanchang University, Nanchang 330006, China

In the article titled “Expression of HIF-2*α* and VEGF in Cervical Squamous Cell Carcinoma and Its Clinical Significance” [1], grant number 81260187 was for a previous project of the author Changshui Xu relating to AIDS and dementia, and it was incorrectly included in the “Acknowledgments” section. The Acknowledgments section should read as follows:

This work was supported by the Youth Science Foundation of the Educational Department of Jiangxi Province (Grant no. GJJ14146), the Cultivating Foundation of Young Scientists (Star of Jinggang) of Jiangxi Province (Grant no. 20153BCB23033), and the Innovation Foundation of the Graduate School of Jiangxi Province (Grants nos. YC2015-S040 and YC2014-S098).

Furthermore, the city of the first affiliation was misspelled and it is corrected above.
